# Prevalence and Associated Clinical Characteristics of Exercise-Induced ST-Segment Elevation in Lead aVR

**DOI:** 10.1371/journal.pone.0160185

**Published:** 2016-07-28

**Authors:** James McKinney, Ian Pitcher, Christopher B. Fordyce, Masoud Yousefi, Tee Joo Yeo, Andrew Ignaszewski, Saul Isserow, Sammy Chan, Krishnan Ramanathan, Carolyn M. Taylor

**Affiliations:** 1 University of British Columbia, Vancouver, British Columbia, Canada; 2 Duke Clinical Research Institute, Durham, North Carolina, United States of America; 3 Clinical Research Unit, Vancouver Coastal Health Research Institute, Vancouver, British Columbia, Canada; 4 National University Heart Centre, Singapore; 5 Division of Cardiology, St Paul’s Hospital, Vancouver, British Columbia, Canada; Medstar Washington Hospital Center, UNITED STATES

## Abstract

**Background:**

Exercise-induced ST-segment elevation (STE) in lead aVR may be an important indicator of prognostically important coronary artery disease (CAD). However, the prevalence and associated clinical features of exercise-induced STE in lead aVR among consecutive patients referred for exercise stress electrocardiography (ExECG) is unknown.

**Methods:**

All consecutive patients receiving a Bruce protocol ExECG for the diagnosis of CAD at a tertiary care academic center were included over a two-year period. Clinical characteristics, including results of coronary angiography, were compared between patients with and without exercise-induced STE in lead aVR.

**Results:**

Among 2227 patients undergoing ExECG, exercise-induced STE ≥1.0mm in lead aVR occurred in 3.4% of patients. Patients with STE in lead aVR had significantly lower Duke Treadmill Scores (DTS) (-0.5 vs. 7.0, p<0.01) and a higher frequency of positive test results (60.2% vs. 7.3%, p<0.01). Furthermore, patients with STE in lead aVR were more likely to undergo subsequent cardiac catheterization than those without STE in lead aVR (p<0.01, odds ratio = 4.2).

**Conclusions:**

Among patients referred for ExECG for suspected CAD, exercise-induced STE in lead aVR was associated with a higher risk DTS, an increased likelihood of a positive ExECG, and referral for subsequent coronary angiography. These results suggest that exercise-induced STE in lead aVR may represent a useful ECG feature among patients undergoing ExECG in the risk stratification of patients.

## Introduction

Exercise stress electrocardiography (ExECG) provides important prognostic and diagnostic information in those with suspected coronary artery disease (CAD).[1] While stress-imaging modalities (Single-photon emission computed tomography, positron emission tomography, cardiac magnetic resonance imaging, stress echocardiography) may provide improved diagnostic accuracy, ExECG possesses the advantages of lower cost, no radiation or chemical exposure, and widespread availability.[2] Guidelines recommend ExECG as the initial diagnostic test for patients with intermediate pre-test probability (and an interpretable ECG).[3,4] Despite these recommendations, the use of ExECG as the initial testing modality in contemporary patients with stable chest pain is remarkably low. Data from the PROMISE trial demonstrated that only 10.2% of patients underwent stress testing without imaging.[5]

Exercise stress electrocardiography can identify high-risk patients in whom referral for invasive coronary angiography is recommended to ascertain the presence of prognostically important CAD.[4,6] Exercise capacity (in metabolic equivalents (METS)), ischemic ST-depression, heart rate recovery, failure to achieve target heart rate, the presence of typical anginal symptoms, and the presence of ST-elevation in lead aVR are variables that have shown to improve risk stratification.[2]

Therefore, ExECG may be more attractive if additional features could be identified that provide incremental diagnostic or prognostic information. Guidelines currently do not recommend using ST-segment changes in lead aVR for test interpretation.[6] However, some smaller studies suggest that exercise-induced ST-segment elevation (STE) in lead aVR may be of significant clinical relevance, being associated with LM and proximal left anterior descending (pLAD) disease.[7–12] The clinical utility of lead aVR extends beyond the ST-segment and prediction of surgical CAD (LM, pLAD, and triple-vessel disease)[13–15]; the presence and magnitude of upright T waves in lead aVR is an independent marker of cardiovascular mortality.[16]

Existing studies exploring the utility of stress-induced STE in lead aVR are limited by their retrospective nature and highly selected patient populations. Furthermore, the baseline prevalence of exercise induced STE in lead aVR in unselected patients is unknown. The aim of this study was to determine the prevalence of exercise-induced STE in lead aVR in consecutive patients referred for ExECG for suspected CAD, and to describe patient characteristics and exercise test parameters associated with this potentially important finding.

## Methods

ExECGs performed between July 1st 2010 and June 30th 2012 at a tertiary care academic center (St. Paul’s Hospital, Vancouver, BC, Canada) were extracted from the exercise test database (MUSE^®^ Cardiology Information System[17]), which captures all treadmill tests performed on-site. All subjects were de-identified and anonymized. The study was approved by the University of British Columbia research ethics board (H12-02210). Only Bruce protocol exercise tests with an interpretable electrocardiogram (ECG) were included. ExECGs with an indication other than for the detection of CAD were excluded. In patients with more than one ExECG performed within the time period of interest, the first ExECG was used. The following variables were obtained for all ExECGs: duration of exercise, maximum METS, maximum ST depression, measurement of ST-segment elevation in lead aVR at rest and at peak stress, slope (upsloping, horizontal, downsloping) of ST-segment in lead aVR at peak stress, reason for termination of ExECG, presence of non-limiting or limiting chest pain, resting ECG interpretation, and test interpretation (negative, equivocal or positive). The slope of STE in lead aVR at peak exercise was manually recorded in all patients and reported as upsloping, horizontal or downsloping. The measurement of the ST-segment in lead aVR at peak stress was computer generated. A random sample of 100 ExECGs were measured using a manual electronic caliper (PixelRuler, Version 2.5, Appthology)[18] to assess computer accuracy. Resting ECGs with left bundle branch block (LBBB), paced rhythm, interventricular conduction delay >120ms, left ventricular hypertrophy, idioventricular rhythm, Wolf-Parkinson-White, exercise-induced LBBB or resting STE >0.5mV in lead aVR were excluded. A Duke Treadmill Score (DTS)[19] was calculated for each patient from the extracted values using the formula: total exercise time in minutes–(5 x ST depression)–(4 x chest pain index).

Exercise-induced STE in lead aVR of 1.0 mm or greater was used to define the presence of STE in lead aVR, as previously defined.[12] All ExECGs with computer measured STE ≥0.75mm were manually examined independently by two cardiologists, blinded to all other exercise test parameters and patient data. A third cardiologist provided consensus reads in cases of disagreement. The ST-segment was measured 80ms from the J point. Only horizontal or up-sloping STE ≥1.0mm in lead aVR was considered significant—downsloping STE in lead aVR was excluded from analysis, as per previous study protocols.[12]

All subjects who underwent cardiac catheterization, percutaneous coronary intervention (PCI) or coronary artery bypass graft (CABG) in the province of British Columbia within six months of ExECG were subsequently identified through the Cardiac Services BC Registry, a comprehensive database that captures all cardiac procedures performed in the province. Rates of significant LM and pLAD disease were determined, as defined by a stenosis ≥50% and ≥ 70%, respectively.[20]

Continuous variables with normal distribution were analyzed by unpaired t-test. Continuous variables with non-normal distribution were analyzed by Mann–Whitney test. Categorical parameters were analyzed by Chi-square or Fisher Exact tests. Two-sided p-values <0.05 were considered significant. Logistic regression analysis was performed to evaluate various risk factors effects on STE in lead aVR. Statistical analysis was performed using a commercially available SPSS v20 and XLSTAT 2014.1.03. Ethics approval was obtained through our local research ethic board.

## Results

**Fig 1:**
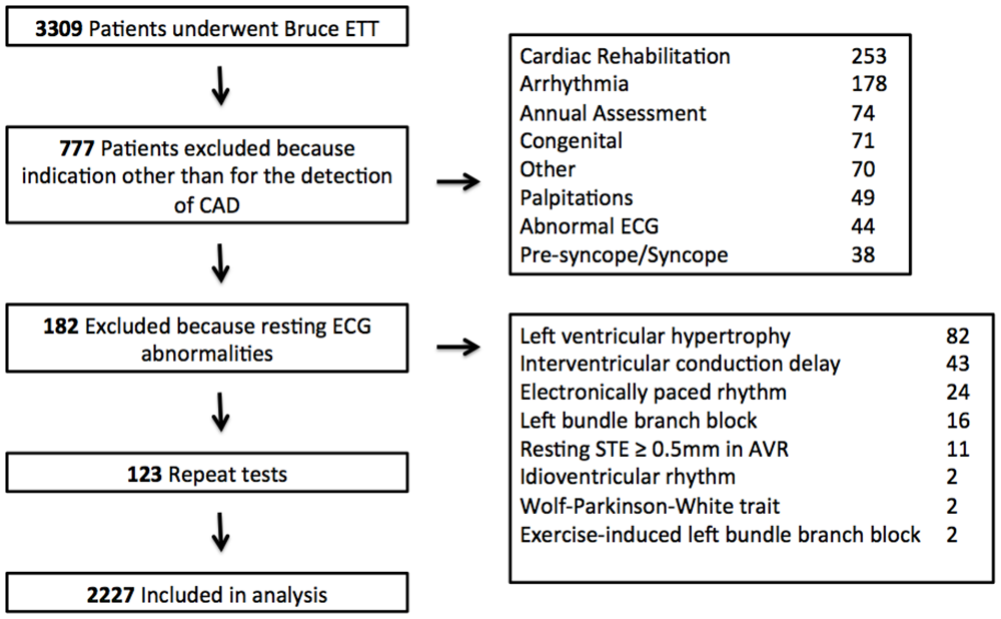
Patient flow diagram. (LVH, left ventricular hypertrophy; IVCD, interventricular conduction delay; LBBB, left bundle branch block).

Between July 1st 2010 and June 30th 2012, the MUSE^®^ database search yielded a total of 3309 ExECGs performed with the Bruce protocol (Table 1). Of those, 777 were excluded because they were performed for an indication other than for the detection of CAD and 182 tests were excluded because of resting ECG abnormalities. Repeat tests (n = 123) were also excluded. A total of 2227 patients were included in the final analysis. Fig 1 demonstrates the patient flow diagram. The mean age of the cohort was 58.4 years with 62.1% male. The correlation between the computer-generated measurement of the ST-segment and the manual measurements made by electronic caliper (r^2^ = 0.97).

**Table 1:** Descriptive characteristics of the cohort (patients referred for exercise stress testing for the indication “detection of coronary artery disease”).

Total patients studied	2227
Age, mean (SD)	58.4 yrs (12.5)
Age, median	59 yrs
Sex, male	62.1%
Exercise duration, in seconds	482.3 (195.6)
Maximum METS achieved	9.6 (4.9)
Chest pain (limiting)	3.0%
Chest pain (limiting or non-limiting)	13.9%
Exercise induced STE in lead aVR ≥ 1 mm	3.4%

STE, ST elevation

Exercise-induced STE ≥0.75 mm and ≥1.0 mm in lead aVR was present in 11.8% (n = 265) and 6.3% (n = 142) of patients, respectively. Of the 142 patients who had STE in lead aVR ≥1.0mm, 75 patients (3.4%) had horizontal or upsloping STE in lead aVR. Clinical and electrographic characteristics among those patients with STE in AVR ≥1.0mm compared to those without are shown in Table 2.

**Table 2:** Exercise test parameters in patients with and without horizontal or upsloping ST-segment elevation ≥1.0mm in lead AVR.

	aVR ≥1mm	aVR<1mm	p-value
	(n = 75)	(n = 2152)	
Age (in years)	63.5	58.3	p<0.01
Metabolic equivalents (METS)	8.9	10.1	p = 0.48
Time in Exercise (min)	7.5	8.0	p = 0.06
Duke Treadmill Score (mean)	-0.2	6.8	p<0.01
Duke Treadmill Score (median)	-0.5	7.0	p<0.01
Low-risk Duke Treadmill Score (≥ +5)	30.7%	72.6%	p<0.01
Moderate-risk Duke Treadmill Score (-10 to +4)	60.0%	27.0%	p<0.01
High-risk Duke Treadmill Score (≤ -11)	9.3%	0.4%	p<0.01
Electrically Positive Test (horizontal or downsloping ST segment depression) (%)	60.2%	7.3%	p<0.01
Right Bundle Branch Block	2.6%	3.7%	p = 0.16
Chest pain (limiting)	5.3%	2.5%	p = 0.06
Chest pain (limiting or non-limiting)	21.3%	13.6%	p = 0.06
Pathological ST depression as reason for test termination	4.3%	0.2%	p<0.01

Maximum METS achieved and duration of exercise were not significantly different in those with exercise-induced STE in aVR vs. those without (8.9 vs. 10.1 METS, p = 0.48) and (7.5 vs. 8.0 min, p = 0.06), respectively. Patients with STE in lead aVR were significantly older than those without STE in aVR, 63.5 vs. 58.3 years, respectively (p<0.01). The DTS differed significantly between the two groups; -0.5 in those with exercise-induced STE in lead aVR vs. 7.0 in those without (p<0.01). Patients with STE in lead aVR (versus those without) were significantly more likely to have a high-risk DTS (≤11; 9.3% vs. 0.4%, p<0.01) and less likely to have a low-risk DTS (≥5; 30.7% vs. 72.6%, p<0.01). In patients with STE ≥1.0mm in lead aVR, 60.2% had an electrically positive ExECG. In contrast, only 7.3% of patients without exercise-induced STE in lead aVR had a positive test (p<0.01).

Limiting chest pain occurred in 5.3% (4/75) of patients with STE in lead aVR ≥1.0mm vs. 2.5% (65/2152) in those without STE in lead aVR (p = 0.06). Furthermore, both limiting and non-limiting chest pain occurred in 21.3% (16/75) of patients with STE in lead aVR ≥1.0mm group vs. 13.6% (294/2152) in those without STE in lead aVR (p = 0.06). Pathological ST depression as a reason for test termination was seen more frequently in the STE in lead aVR ≥1.0mm group compared to those without STE in lead aVR (4.3% vs. 0.2%, p<0.01).

There was no significant association between the presence of right bundle branch block (RBBB) and STE in aVR. Right bundle branch block existed in 81 (3.8%) of the total patients and STE in lead aVR ≥1.0mm only occurred in two of those 81 patients with RBBB (p = 0.16).

Seventy-nine of 2227 patients (3.5%) in the study underwent cardiac catheterization within six months of ExECG. Patients with exercise-induced STE in lead aVR were more likely to undergo cardiac catheterization, compared to those without STE in lead aVR (12% (9/75) vs. 3.3%; p<0.01, OR = 4.2)(Table 3). Amongst patients undergoing cardiac catheterization, significant left main (11.1% vs. 7.1%, p = 0.58) and pLAD (44.4% vs. 20.0%, p = 0.20) CAD was common, but was not significantly different amongst those with exercise-induced STE in lead aVR versus those without, respectively (Table 4). Of importance, the majority of patients with exercise induced STE in lead aVR (64/75) were not referred for invasive angiography within 6 months following ExECG.

**Table 3:** Clinical referral for cardiac catheterization within six months of exercise testing.

	Total (n = 2227)	aVR≥1mm (n = 75)	aVR<1mm (n = 2152)
Number of patients referred for cardiac catheterization within six months of exercise testing n(%)	79 (3.5%)	9 (12.0%)	70 (3.3%)

**Table 4:** Coronary anatomy amongst those undergoing clinically indicated cardiac catheterization.

	Exercise-induced STE in lead aVR ≥1mm	Exercise-induced STE in lead aVR<1mm	p-value
	n = 9	n = 70	
LM CAD	11.1%	7.1%	p = 0.58
pLAD CAD	44.4%	20.0%	p = 0.20
LM or pLAD CAD	55.6%	27.1%	p = 0.12

CAD, coronary artery disease; LM, left main; pLAD, proximal left anterior descending

## Discussion

Among consecutive patients referred to a tertiary care center for the indication of diagnosing CAD, exercise induced STE in lead aVR was a common finding, occurring in 6.3% of subjects. The prevalence of horizontal or upsloping STE in lead aVR was 3.4%. Furthermore, STE in lead aVR was associated with increased age, a lower DTS, greater chance of a positive test and increased rates of subsequent cardiac catheterization. These findings suggest that STE in lead aVR is common among patients with stable chest pain. While this finding may represent a high-risk feature, the majority of patients with exercise induced STE in lead aVR were not referred for early invasive angiography.

ST-segment elevation in lead aVR in the setting of acute coronary syndrome[21–25] and ExECG[7,10–12] has been shown to yield important anatomical and clinical information. Amongst high-risk patients (DTS<-11), the reported prevalence of STE in aVR during ExECG has been reported to be as high as 62.5% in those selected groups.[11] However, until now, the prevalence of STE in lead aVR amongst unselected patients referred for the clinical indication of detection of CAD has been unknown.

Lead aVR has historically been believed to merely provide reciprocal information from the oppositely oriented left lateral leads. However, lead aVR is also postulated to behave as a ‘‘pseudo-intracavitary” lead, detecting interventricular amplitudes orientated towards the cavity of the heart. Therefore, lead aVR may be able to detect “global left ventricular ischemia” as would be expected with significant LM or pLAD disease. The presence of STE in lead aVR in the setting of acute coronary syndromes has been predictive of significant LM or pLAD disease.[13,21–28] Furthermore, STE in lead aVR has been shown to be a negative prognostic indicator as it is associated with increased in-hospital death and mortality at 1, 3, 6 and 12 months as well as a higher incidence of heart failure.[21,25] It also adds prognostic value to low and intermediate GRACE scores.[25]

This study is not without limitations. First, inherent bias may exist due to the retrospective nature of this large clinical database. Nonetheless, the ‘real-world’ consecutive nature of this population may have broad applicability. Second, efforts were made to maintain the integrity of the patient population by excluding patients who underwent ExECG for other reasons (valvular heart disease, arrhythmia, etc.) than to assess for CAD. However, concomitant conditions amongst our population could not be entirely ruled out. Third, since few patients subsequently underwent cardiac catheterization, we are underpowered to detect meaningful associations between ExECG results and coronary anatomy. However, the intent of the study was not to correlate STE in aVR with coronary anatomy. Establishing an individual’s coronary anatomy without clinical indication is unjustified, and hence we only have anatomical data on a limited number of patients who had a recommended indication for cardiac catheterization. The goal of this study was to document the prevalence and associations in a real world population. Finally, long-term clinical outcomes were not captured in this dataset.

This study establishes the prevalence of horizontal or upsloping STE ≥1.0mm in lead aVR patients referred with the indication of diagnosing CAD with exercise stress testing as 3.4%. Patients with exercise induced STE in aVR tended to be older, have a higher risk DTS, positive exercise stress test results and were referred for early invasive angiography more often. These findings suggest that STE in lead aVR is relatively common among patients with stable chest pain, and is associated with higher risk features. The presence of exercise induced STE in lead aVR may represent an additional feature for risk stratification amongst patients undergoing ExECG.
